# Effects of Cadmium Bioavailability in Food on Its Distribution in Different Tissues in the Ground Beetle *Pterostichus oblongopunctatus*

**DOI:** 10.1007/s00128-019-02679-x

**Published:** 2019-07-24

**Authors:** Agnieszka J. Bednarska, Zuzanna M. Świątek, Anna Maria Labecka

**Affiliations:** 1 Institute of Nature Conservation, Polish Academy of Sciences, Mickiewicza 33, 31-120 Kraków, Poland; 2 Institute of Environmental Sciences, Jagiellonian University, Gronostajowa 7, 30-387 Kraków, Poland

**Keywords:** Metal, Digestive tract, Malpighian tubules, Carabidae

## Abstract

In most laboratory studies with oral exposure of terrestrial invertebrates to metals an artificial food, which is easy to handle, is used. The bioavailability of metals from this artificial food may, however, be much higher than from more field relevant food sources. Such differences may affect toxicokinetic processes in different tissues. To test the effect of bioavailability of Cd in food on Cd toxicokinetics and internal distribution in terrestrial invertebrates, we performed the experiment using the ground beetle *Pterostichus oblongopunctatus* exposed to Cd via food differing in their soluble Cd pool. We showed that in carabids Cd accumulation and elimination pattern in different tissues is not governed by the metal availability in food.

Ground beetles (Carabidae: Coleoptera) have been shown to be relatively resistant to cadmium, zinc and other metals. They survive in areas highly contaminated with metals (Butovsky 2011) and laboratory experiments have revealed that during prolonged metal exposure they are able to maintain metal concentration in their bodies unchanged (Kramarz 1999) or to even decrease it (Bednarska et al. 2011). In general, at high levels of metal exposure three strategies aid survival: reducing metal uptake, eliminating metal ions in urine and/or faeces, and sequestration of metal ions by proteins and other ligands and/or incorporation into inorganic crystalline concretions (granules) which prevent metals from interfering with biochemical pathways (Hopkin 1989). The most important regulatory organ for metals in terrestrial invertebrates is the gut, which is involved in the uptake, transport, storage and excretion of metals in insects (Hopkin 1989). Nevertheless, the detoxification of metals is not limited to this organ. The study on the housefly, *Musca domestica* (Sohal et al. 1976) or silkworm larvae, *Bombyx mori* (Suzuki et al. 1984) highlighted the importance of Malpighian tubules in the detoxification of metals. The presence of intracellular granules—important in storing metal ions—in Malpighian tubules has been also demonstrated for five species of Coleoptera (Hopkin 1989). Zinc, copper and cadmium deposition was also documented in the exoskeleton of grasshoppers and carabid beetles; this was interpreted as a strengthening mechanism (e.g. the hardening of invertebrate mandibles; Hillerton and Vincent 1982) or as a detoxification pathway through moulting (Lindqvist et al. 1995).

The amount of metals assimilated from food by invertebrates in terrestrial ecosystems are controlled by two factors: the concentration of ‘available’ metals in the food and the physiological mechanisms which they possess for uptake and excretion of metals (Hopkin 1989). The same metal present in food at the same concentration may be assimilated at different rates, as the rates of assimilation of metals in animals strongly depend on the ligands to which the elements are bound in the food. For example, lipid-soluble complexes are usually very stable and unlikely to release toxic free metal ions (Batley 1989). The chemical form of a metal (i.e., speciation) in food determines how easily the metal is converted in the gut lumen to a form available for assimilation (Hopkin 1989). Metals are present in food in different forms, and the two main pools are metal ions bound to the solid phase and the soluble metal pool. The ratio between these pools can differ depending on the food type and may affect the rate of assimilation and/or elimination of the metal inside the body. Surprisingly, very few experimental studies are available addressing the toxicokinetics of metals in different compartments in response to the chemical form of a metal (i.e., speciation) in food (Bednarska et al. 2017).

In most laboratory studies with oral exposure, a food artificially spiked with the metal of interest is usually used, so the metal is present in the food in a soluble form (i.e., ionic form). The availability of the metal from such an artificially contaminated food for assimilation by a consumer (predator) is considered much higher than from the food consumed by an animal in the field since a prey sequesters at least some accumulated Cd in a form not available for consumers [e.g. through the binding of metals to heat-stable proteins located mainly in the cytosol and the formation of distinct inclusion bodies (granules)]. For example, *Tenebrio molitor* larvae reared on Cd contaminated medium sequestered ca. 30% of Cd in the fraction which is important for the transport of metals to higher trophic levels in a food web (soluble pool, available to a predator) and the contribution of Cd in the granules to the total Cd amount was 30%–40% (Bednarska and Świątek 2016). Because metals bound to insoluble fractions, i.e. cellular debris, exoskeleton, and metal granules contained in prey body, are considered not readily available for assimilation by a consumer (Wallace et al. 2003; Vijver et al. 2004), it is not clear to what extent the toxicokinetic parameters derived from laboratory experiments based on metal-spiked food are indicative of the field situation. Although there are some data on the effect of chemical partitioning of metals in exposure medium on their bioavailability (Peijnenburg et al. 1999), toxicokinetics and internal sequestration (Vijver et al. 2004) for invertebrates exposed via soil and soil solution, similar data for carnivorous insects exposed via food are scarce. One of the few such studies, in which the effect of subcellular partitioning of Cd in plants on the transfer of Cd to the isopod *Porcellio dilatatus* was studied, showed that the subcellular Cd distribution in food may have an important impact on the trophic transfer of Cd to the consumer (Monteiro et al. 2008). Similarly, our recent study on the ground beetle *Pterostichus oblongopunctatus* indicated that Cd transfer in the food web depends on the speciation of the metal in the food (Bednarska et al. 2017). However, regardless of the bioavailability of Cd in food (food naturally vs. artificially contaminated with Cd), we found no effect of the type of food on Cd sequestration kinetics over different subcellular fractions in *P. oblongopunctatus* (Bednarska et al. 2017).

To better understand the effect of bioavailability of Cd in food on toxicokinetics and internal distribution of Cd in terrestrial invertebrates, we performed an experiment similar to that of Bednarska et al. (2017), in which *P. oblongopunctatus* were offered the same type of food differing only in the soluble metal pool, but this time we focused on anatomical rather than subcellular distinction between compartments. The kinetics of Cd was followed in three different compartments—the digestive tract, Malpighian tubules and the rest of the body.

## Materials and Methods

The experiment was performed on the ground beetle *P. oblongopunctatus*. Adult males were collected with pitfall traps at an uncontaminated site near Krakow, Southern Poland, in April 2015. They were kept for 4 weeks to acclimatise to laboratory conditions as described by Bednarska et al. (2017). Before starting the experiment, the beetles were weighed to the nearest 0.0001 g (Radwag AS/C/2, Poland) and placed individually in 30-mL plastic boxes filled to ca. 1/4 with moistened sand (Grudzeń Las Sp. z o. o., Poland) and with a piece of clay pot placed in each box to provide a shelter for the beetles. The beetles were randomly allocated to two different Cd treatments and were fed with metal contaminated food for 28 days (uptake phase), and afterwards an uncontaminated food was offered for another 12 days (decontamination phase). The food was either ‘naturally’ or artificially contaminated with Cd. The ‘naturally’ contaminated food (Cd-N) was made of ground mealworms *T. molitor* reared for two weeks on the flour spiked with CdCl_2_ × 2.5 H_2_O (Avantor Performance Materials, Poland), and then mixed with ground apple (9:1). The food artificially contaminated with Cd (Cd-A) was made of ground mealworms reared on uncontaminated flour and then spiked with the metal-salt solution and mixed with ground apple (9:1) to obtain the same Cd concentration as the concentration measured in the ‘naturally’ contaminated food. Thus, the same type of food, differing only in the chemical partitioning of metal in the food, was used: Cd concentration in the Cd-spiked diet (Cd-A) was 308 ± 12 mg kg^−1^ dry weight (mean ± standard deviation, SD) or the equivalent of 117% of the Cd concentration in a ‘naturally’ contaminated diet, which was 264 ± 10 mg kg^−1^, and the difference in Cd concentration between Cd-A and Cd-N food was statistically nonsignificant (Bednarska et al. 2017). Our earlier study showed that *T. molitor* larvae reared on flour contaminated with Cd sequestered ca. 30% of Cd in the fraction important for transport of metals to higher trophic levels (Bednarska and Świątek 2016). Thus, the bioavailability of Cd to a predator (here *P. oblongopunctatus*) was surely higher in artificially spiked *T. molitor* larvae than in the same larvae reared on Cd-contaminated medium, as in the first case the whole Cd was present in the highly bioavailable ionic form. The control treatment with uncontaminated food (average Cd concentration of 0.17 ± 0.0015 mg kg^−1^ dry weight) offered throughout the experiment was also included. Ten beetles were sampled before starting the exposure (day 0) and then three individuals per treatment were sampled after 2, 4, 6, 8, 16, 20, 24 and 28 days of the uptake phase, and at days 30, 34 and 40 days (decontamination phase, also described in the literature as the elimination phase). The sampled beetles were starved for 24 h to reduced uncertainty in Cd and body weight measurements, washed with deionized water to remove all remnants of food from their body surface, weighed to the nearest 0.0001 g and prepared for anatomical analysis.

After cutting-off of the head and elytra, the beetle thorax was dissected in 1 × PBS solution (Avantor Performance Materials, Poland) under a stereoscopic microscope (SZ40, Olympus, Japan). The digestive tract and the Malpighian tubule system were removed from the body. Additionally, the digestive tract was cleaned with a 1 × PBS-filled syringe-needle. The rest of the body, including the soft and exoskeletal parts, provided the third compartment. All samples were stored in 1 × PBS at 4 °C until they were analysed for metal concentrations.

To analyse the Cd concentration in the three compartments, the tissue samples in 1 × PBS buffer were placed in 2 mL glass tubes and dried at 105 °C for 24 h before digesting in 300 μL of boiling HNO_3_. Samples with 1 × PBS buffer only were used to control for possible contamination during the dissection procedure. After complete digestion, the excess of acid was evaporated and the samples were diluted to 1 mL with 0.2% HNO_3_ (69.0%–70.0%, INSTRA-Analysed, Baker, Germany). Cadmium concentration in each compartment was analysed with a graphite furnace atomic absorption spectrophotometer (Perkin-Elmer AAnalyst 800; detection limit: 0.024 µg L^−1^) as described by Bednarska et al. (2017). To check the analytical precision, three blanks (acid only) and three samples of a reference material (fish liver—C*ertified Reference Material Dolt-4 Dogfish Liver* or C*ertified Reference Material Dolt-5 Dogfish Liver*, National Research Council of Canada) were run with the samples. The measured Cd concentration in the reference material was within ± 6% of the certified value. The results were not corrected for recovery. The amount of the metal measured in each anatomical fraction was normalised by dividing it by the beetle wet weight (body weight normalised level, BWNL) and, thus, the levels of Cd in different fractions were expressed in µg kg^−1^ beetle fresh weight.

The pattern of changes in Cd levels over time was analysed by fitting the classic one-compartment toxicokinetics model to metal BWNL levels in each anatomical compartment and treatment separately, using the equations and procedures described by Skip et al. (2014). Because the temporal pattern in the BWNL of Cd did not allow for testing for possible differences in kinetic parameters between treatments, the effects of the food type (Cd-N vs. Cd-A) and the duration of exposure on BWNLs of Cd in each anatomical compartment were additionally tested separately for the uptake and decontamination phases using a two-way ANOVA with beetles body weight at sampling time as a covariate. The BWNLs of metals in each fraction were rank-transformed prior to ANOVA (Zar 1999). Day 0, which was common for both treatments, was excluded from the ANOVA to allow for testing interactions between the factors. After testing for both main factors and their interaction for significance (p ≤ 0.05), the non-significant interaction was removed from the model. Statistically significant differences were further analysed using Fisher's least significant difference (LSD) test for the post hoc comparison of means. Similar analysis was done to test the effect of the food type (Cd-N vs. Cd-A) and the duration of exposure on Cd proportion (in percentage) in each anatomical compartment. The percentage of metal was calculated by relating the amount of metal retrieved from specific anatomical compartment to the total amount of the metal (i.e. the sum of metal amounts in all the studied compartments) in the organism. Prior to ANOVA, the arc sine of the square root transformation was performed for percentages of metals in each fraction (Zar 1999).

One-way ANOVA was used to verify the ability of the beetles to eliminate Cd approaching the initial metal levels by comparing the levels at days 0 and 40 separately for each treatment (i.e. Cd-N, Cd-A and control) and compartment, and for comparison between days 0 and 28 and days 28 and 40 for each treatment and compartment. One-way ANOVA with treatment as the explanatory factor was also used to check for differences in metal levels between treatments at the end of the experiment. The contribution of metal (in percentage) to each anatomical compartment was similarly analysed. All statistical analyses were performed using the Statgraphics Centurion XVI program (StatPoint Technologies, Inc., USA).

## Results and Discussion

In most laboratory experiments with metal exposure via food, an artificial food spiked with the metal salt solution is used. However, using artificial food may lead to significant inaccuracies when results obtained in laboratory experiments need to be extrapolated to the field. For example, in their toxicokinetic experiment on Zn and Cd kinetic in the ground beetle *P. oblongopunctatus* Lagisz et al. (2005)*,* observed metal elimination already in the uptake phase, which did not allow them to fit the one-compartment model to their data. The results obtained by Lagisz et al. (2005) were especially surprising because the uptake and elimination kinetics consistent with expectations of the classic one-compartment model were found previously for the same metals in the closely related carabid *P. cupreus* (Kramarz 1999). The differences observed between the results for *P. oblongopunctatus* (Lagisz et al. 2005) and for *P. cupreus* (Kramarz 1999) might be due to the use of different medium to feed the beetles: Lagisz et al. (2005) used artificial food (dried chicken meat mixed with metal-salt solution), whereas Kramarz (1999) fed the beetles with housefly larvae reared on artificial medium contaminated with metals. The latter method seems to better resemble the actual feeding habits of ground beetles, and the bioavailability of metals might differ between these two diets. In the present study, we used the same type of food with similar Cd concentrations but differing in the chemical partitioning of the metal: the soluble Cd pool available for *P. oblongopunctatus* was surely higher in artificially spiked *T. molitor* larvae than in larvae reared on the Cd contaminated medium. To prepare the food ‘naturally’ contaminated with Cd, larvae reared for 2 weeks on the flour contaminated with Cd at 374 ± 2 mg kg^−1^ were used (Bednarska et al. 2017) and our earlier study showed that such *T. molitor* larvae (i.e., larvae reared for 2 weeks on flour contaminated with Cd at 380 ± 36 mg kg^−1^, so at almost identical concentration as in this study) sequestered only 27% of Cd in the fraction important for transport of the metal to higher trophic levels and the percentage of Cd in granules was 50% of the total Cd level in the body (Bednarska and Świątek 2016). Although the Cd concentration in the potential food of ground beetles - such as plants and small invertebrates - is usually lower than those used in our study (e.g., internal Cd concentrations for eight taxonomic groups of terrestrial invertebrates reported by Heikens et al. (2001) range from ca 1 up to 110 mg kg^−1^), some extreme cases have been reported, e.g. plants with Cd up to 560 mg kg^−1^ (Kabata-Pendias and Mukherjee 2007). Thus, at least theoretically, the beetles can be exposed to Cd concentrations even higher than those used in our study. The concentration of Cd in food used in our study did not affect survival of the beetles (p = 0.7; logrank test) and the mortality did not exceed 20% in any of the treatments, which allowed us to dissect 3 beetles per sampling day per treatment in all cases but two (day 8 in control and day 20 in Cd-N, when only 2 individuals survived the 24-h starvation). If more than three individuals survived till the end of the experiment, more were sampled and this was the case for the Cd-N treatment for which 9 individuals were sampled, as well as for the Cd-A and control treatments for which 4 individuals were sampled at day 40. The initial wet weight of the beetles was 0.057 ± 0.0066 g (mean ± SD) for Cd-N treatment, 0.058 ± 0.0061 g for Cd-A treatment and 0.056 ± 0.0067 for control with no significant differences between treatments (p = 0.3, ANOVA), and all the beetles sampled for anatomical fractionation gained weight during the study.

**Fig. 1:**
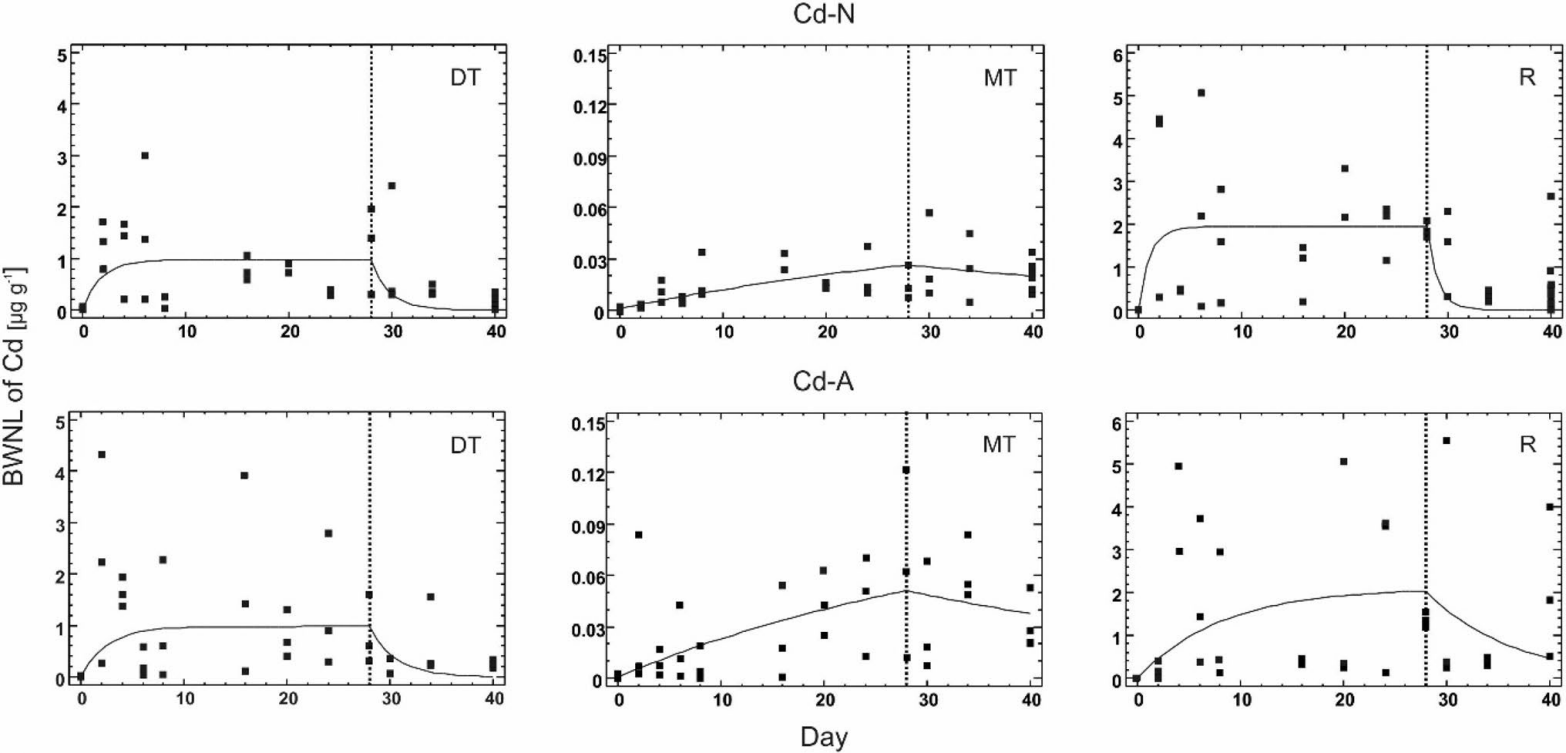
Cadmium toxicokinetic described by the one-compartment model in different anatomical compartments (*DT* digestive tract, *MT* Malpighian tubules, *R* all other tissues) of the ground beetle *Pterostichus oblongopunctatus* exposed via food made of ground mealworm (*Tenebrio molitor*) larvae, reared either on Cd contaminated medium (Cd-N, upper panel) or on artificially spiked after grinding with CdCl_2_ solution (Cd-A, lower panel). The solid lines indicate the fitted model to body weight normalized level (BWNL) of Cd and the vertical broken lines indicate the day of transferring the animals to control food

The obtained results suggest that patterns of uptake and elimination of Cd in different anatomical compartments of *P. oblonopunctatus* do not differ among the food types. It has to be stressed, however, that although the one-compartment model with two phases could be fitted to the BWNLs of Cd in different anatomical compartments and treatments (Fig. 1), the fit was poor (R^2^ ranged from 0% to 32%) and kinetic parameters were nonsignificant (i.e. their confidence intervals covered 0). In fact, the only significant estimate of *k*_*A*_ was for Cd-N (0.000004 day^−1^) and Cd-A (0.000007 day^−1^) in Malpighian tubules (Table 1). The high variance in Cd levels in all compartments throughout the experiment probably indicates the inter-individual differences in Cd handling, what was observed already earlier for this species (Bednarska et al. 2017; Bednarska and Stachowicz 2013; Lagisz et al. 2005) and other invertebrates under many exposure scenarios (Spurgeon et al. 2011).

**Table 1:** Actual Cd levels in the food (mean ± SD) and the estimated toxicokinetic parameters (*k*_*A*_—assimilation and *k*_*E*_—elimination rate constants) with asymptotic 95% confidence intervals for the one-compartment model for different anatomical compartments (*DT* digestive tract, *MT* Malpighian tubules, *R* all other tissues) in the ground beetle *Pterostichus oblongopunctatus* exposed via food made of ground mealworm (*Tenebrio molitor*) larvae, either reared on Cd contaminated medium (Cd-N) or artificially spiked after grinding with CdCl_2_ (Cd-A); *C*_0_, *C*_28_ and *C*_40_—BWNL of Cd (µg kg^−1^) or percentage (%) of Cd in each anatomical compartment at the start and at the end of the uptake and decontamination phases, respectively

Treatment	Cd in food	Fraction	*k*_*A*_	*k*_*A*_	*R* ^2^	*C*_0_	*C*_28_	*C*_40_	*C*_0_	*C*_28_	*C*_40_
	(mg kg^−1^)		(day^−1^)	(day^−1^)	%	(µg kg^−1^)			(%)		
Control	0.17 ± 0.0015	DT	–	–	–		7.8 ± 9.22^a^	1.9 ± 1.49^a^		22.0^a^	13.8^a^
		MT	–	–	–		0.9 ± 0.28^a^	2.2 ± 2.77^a^		3.9^a^	5.0^a^
		R	–	–	–		58 ± 66.7*^a^	20 ± 14.0^a^		74.1^a^	81.2*^a^
Cd-N	264 ± 10	DT	0.002 (*NS*)	0.58 (*NS*)	20	5.1 ± 6.15	1219 ± 841*^b^	193 ± 145.6*^#b^	43.9	35.4^a^	30.2^a^
		MT	0.000004	0.02 (*NS*)	32	1.0 ± 0.35	16 ± 9.9*^b^	21.0 ± 7.3*^b^	13.4	0.5*^a^	4.1*^#a^
		R	0.007 (*NS*)	0.96 (*NS*)	24	6.0 ± 4.99	1892 ± 163*^b^	654 ± 792.7*^#b^	42.7	64.1^a^	65.7^a^
Cd-A	208 ± 12	DT	0.001 (*NS*)	0.43 (*NS*)	11		839 ± 675.1*^b^	234 ± 78.7*^#b^		33.5^a^	17.4^a^
		MT	0.000007	0.02 (*NS*)	32		65 ± 54.7*^b^	30 ± 15.4*^b^		3.4^a^	1.9^a^
		R	0.0008 (*NS*)	0.12 (*NS*)	7		1363 ± 171*^b^	2114 ± 1757*^b^		63.1^a^	80.7^a^

NS nonsignificant (95% confidence interval for the parameters covers zero)

^*^Significant differences (p ≤ 0.05) in Cd concentration (µg kg^−1^ wet weight) or Cd percentage (%) between day 0 (common for all treatments) and day 28 or between day 0 and day 40 for the particular compartment

^#^Significant differences (p ≤ 0.05) in Cd concentration (µg kg^−1^ wet weight) or Cd percentage (%) between day 28 and 40

^a,b^The lowercase letters means significant differences (p ≤ 0.05) in Cd concentration (µg kg^−1^ wet weight) or Cd percentage (%) between treatments (control, Cd-N, Cd-A) for the particular compartment

Cadmium could be detected in each compartment, i.e. the digestive tract, Malphigian tubules and the rest of the body, but its level in the tissues did not depend on the metal availability in the food as no significant effect of the food type (Cd-N vs. Cd-A) on BWNL of Cd in any anatomical compartment (p ≥ 0.16) was found in the beetles sampled in the uptake phase (0 < day ≤ 28). Also, no effect of the day of exposure on BWNL of Cd was found for the studied compartments (p ≥ 0.08) in the uptake phase. Neither food type (Cd-N vs. Cd-A) (p ≥ 0.13) nor time (p ≥ 0.14) was significant for any anatomical compartment in the decontamination phase (day > 28).

BWNL of Cd in each fraction was significantly higher at day 28 relative to day 0 for both Cd-A and Cd-N treatments (p ≤ 0.0001), but the beetles did not eliminate Cd to background levels during the decontamination phase (Table 1). The levels of Cd in both Cd treatments were higher at the end of the elimination phase (day 40) than before the exposure (day 0) for all studied compartments (p ≤ 0.0001, Table 1). Moreover, the Cd-N beetles had significantly lower Cd concentration at day 40 than at day 28 in the digestive tract and the rest of the body (p ≤ 0.05), but for Cd-A similar differences were only found for the digestive tract (p = 0.039). The Cd levels in all compartments at the end of the experiment were significantly lower in the control beetles than in both Cd treatments (p ≤ 0.001), which did not differ from each other (Table 1).

Those individuals for which data on metal levels were not available for all three compartments were excluded from the statistical analysis of metal proportions (percentage) in each compartment. This was the case for 4 out of 114 sampled beetles (Cd-N day 4, Cd-S day 4, control day 0 and day 34).

**Fig. 2:**
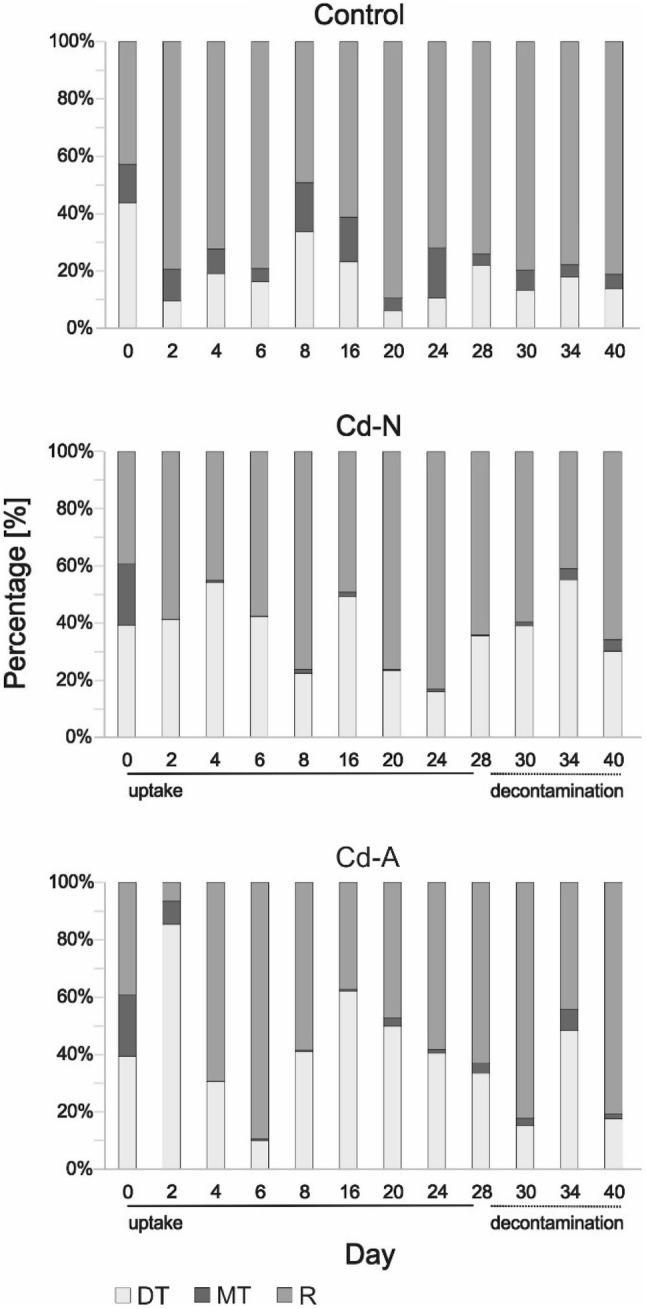
Overall patterns of Cd distribution [%] over different anatomical compartments (*DT* digestive tract, *MT* Malpighian tubules, *R* all other tissues) in the ground beetle *Pterostichus oblongopunctatus* exposed via food made of ground mealworm (*Tenebrio molitor*) larvae, either reared on Cd contaminated medium (Cd-N) or artificially spiked after grinding with CdCl_2_ solution (Cd-A) and in beetles not exposed to Cd (control). The vertical solid line indicates the uptake phase and the broken line indicates the decontamination phase

The cadmium contribution to different anatomical compartments during the uptake and decontamination phases is shown in Fig. 2. In the beetles sampled in the uptake phase (0 < day ≤ 28), two-way ANOVA revealed no significant effect of food type (Cd-N vs. Cd-A, p ≥ 0.2) or day of exposure (p ≥ 0.3) on the percentage of Cd found in any anatomical compartment. Also, in the decontamination phase (day > 28) no effect of Cd treatment (p ≥ 0.6) on the proportion of Cd in any anatomical compartment was found. The decontamination time was significant only for the rest of the body (p = 0.044), with body mass as a covariate at p = 0.005.

There were no differences in the percentage of Cd accumulated in the digestive tract before (day 0) and at the end of the experiment (day 40) in both Cd treatments (p ≥ 0.1). However, the percentage of Cd in the Malpighian tubules was higher at day 0 than at day 40 in the Cd-N beetles (p = 0.027), while in the rest of the body it was lower at day 0 than at day 40 in the Cd-A treatment (p = 0.048). The percentage of Cd in all the studied compartments, but one (i.e., the Malpighian tubules of Cd-N beetles, p = 0.024) did not differ between day 0 and day 28 for both Cd treatments (p ≥ 0.1). Moreover, in all the treatments no significant differences between day 28 and day 40 were found for Cd proportion in the gut and the rest of the body (p ≥ 0.25), while in Malpighian tubules a higher percentage of Cd at day 40 than at day 28 was found in Cd-N beetles (p = 0.04). The percentage of Cd at the end of the decontamination phase did not differ between the treatments (control, Cd-A and Cd-S) in any compartment (p ≥ 0.4).

At the end of the uptake phase, ca. 30% of Cd was accumulated in the beetles’ digestive tract. For comparison, in the alimentary canal of the flesh fly larvae (*Boettcherisca peregrine*) Wu et al. (2009) found ca. 67% of total Cd content and in the Malpighian tubules—ca. 2.8% of the total body Cd content. The authors also reported Cd concentration in the Malpighian tubules, 27.5 mg/g dry weight, which was the highest among the studied tissues. Although we cannot say at which concentrations beetles accumulated Cd in the Malpighian tubules (as our study was not designed to measure this), the proportion of Cd found in the Malpighian tubules of Cd-exposed beetles, 0.5%–3.4%, was similar to that found by Wu et al. (2009). Because the mass of Malpighian tubules is only a small fraction of the total body mass, the concentration of Cd in Malpighian tubules was probably higher than in the rest of the body, which indicates the important role of the Malpighian tubules in the storage and distribution of metals in the ground beetles and no effect of metal bioavailability in the food on these processes. The epithelium of the intestinal tract is the first barrier against assimilation of toxicants into the organism but metals readily cross the gut epithelium into the hemolymph and are then transported into other tissues (Leonard et al. 2009). A few studies highlight the fact that exposure to environmental contaminants via contaminated food may cause sublethal effects on the Malpighian tubules (Giglio and Brandmayr 2017). However, the ultrastructural analysis of the Malpighian tubules of the ground beetle *Carabus lefebvrei* exposed to soil contaminated with different metals showed that potentially toxic metals are safely stored in intracellular compartments in an insoluble and physiologically inactive form (Talarico et al. 2014). To conclude, we have shown that in carabids Cd accumulation and elimination pattern in different tissues is not governed by the metal availability in food.
